# Incidence and seasonality of major ocular trauma: a nationwide population-based study

**DOI:** 10.1038/s41598-020-67315-9

**Published:** 2020-06-22

**Authors:** Jin-woo Kwon, Moon Young Choi, Jung Min Bae

**Affiliations:** 1 Department of Ophthalmology and Visual Science, St. Vincent’s Hospital, College of Medicine, The Catholic University of Korea, Seoul, South Korea; 2 Department of Dermatology, St. Vincent’s Hospital, College of Medicine, The Catholic University of Korea, Seoul, South Korea

**Keywords:** Risk factors, Health occupations, Epidemiology

## Abstract

We designed this study to identify the epidemiological characteristics and trends of various types of ocular trauma in the population of the Republic of Korea. We conducted a nationwide, population-based, cross-sectional study using the Korean National Health Insurance claims database for January 2010 to December 2018. We compiled the monthly numbers of patients diagnosed with hyphema and those who received open reduction surgery due to orbital blowout fracture (BOF), primary closure of the cornea or sclera (PCCS), or intraocular foreign body (IOFB) removal. We obtained annual and monthly incidence rates, and differences according to age, sex, yearly trends, and seasonal variations. The incidence rate (per 100,000 person-years) was high in the order of hyphema (18.43), BOF (11.58), PCCS (1.99) and IOFB removal (0.39). Male predominance was evident in all types of major ocular trauma, but the age distribution varied with the type: hyphemas were most prevalent at 10–14 years of age, BOFs at 25–29 years of age, and open globe injuries (OGIs) at age 60 and older. Although all types of trauma showed significant seasonality, hyphemas (amplitude: 174.81) and BOFs (23.17) showed higher amplitudes compared to OGIs (PCCS: 11.96; IOFB removal: 6.72). While the incidence of blunt trauma injuries, including hyphemas and orbital BOFs, decreased steadily from 2010 to 2018, that of OGIs showed no remarkable change.

## Introduction

Ocular trauma is an important cause of visual impairment^1–3^, and the World Health Organization has reported that globally 55 million people experience serious ocular trauma every year^4^. Depending on the type and severity of the trauma, not only the cost for treatments could be high, but also it could remain irreversible poor visual impairments following the injury^5^. Additionally, blindness under the age of 20 is predominantly due to ocular trauma and its socioeconomic cost is very large^2, 6–8^.

The eyeball injury is classified as either closed globe injury or open globe injury (OGI)^9–11^. The OGI, defined as full thickness wound in cornea or sclera, is classified as rupture and laceration. Corneal or scleral laceration is further classified as perforation, penetrating injury, and intraocular foreign body (IOFB)^9^. Usually, the OGIs require primary closure of the cornea or sclera (PCCS) or surgery of IOFB removal.

Epidemiological studies identifying trends or causes of ocular trauma are essential for establishing appropriate prevention measures. However, previous reports have limitations such as targeting a specific type of ocular trauma^12^, analyzing only emergency visits^1, 2^, small populations^3^, or including a limited number of institutions^11^. In the present study, we sought to identify the epidemiological features of major types of ocular trauma, including closed globe injuries such as hyphemas and orbital blowout fractures (BOFs) and OGIs, based on the Korean National Health Insurance (NHI) claims database.

## Results

### Hyphema

**Figure 1:**
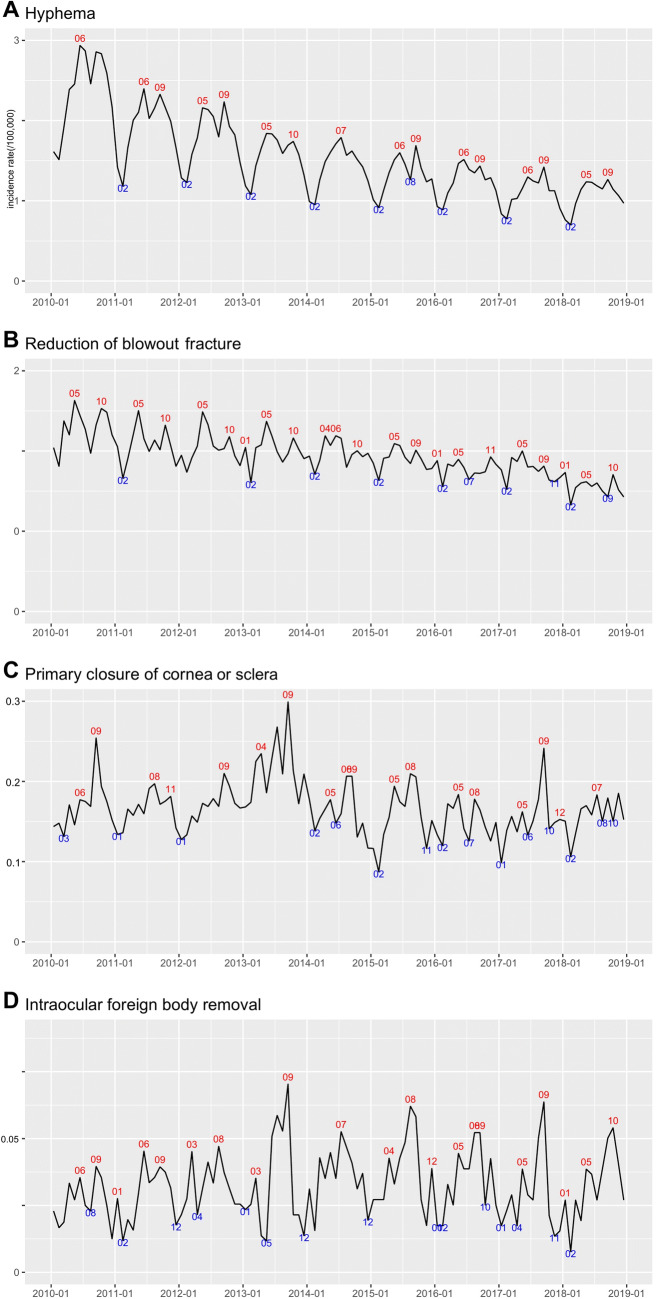
Seasonal variation in different types of eye trauma with monthly incidence rates, 2010–2018. Compared to open globe injuries (**C**, **D**), hyphemas (**A**) and orbital blowout fractures (**B**) showed more prominent seasonality. Additionally, the incidence rates of hyphemas (**A**) and orbital blowout fractures (**B**) decreased annually, but that of open globe injuries (**C**, **D**) did not.

**Figure 2:**
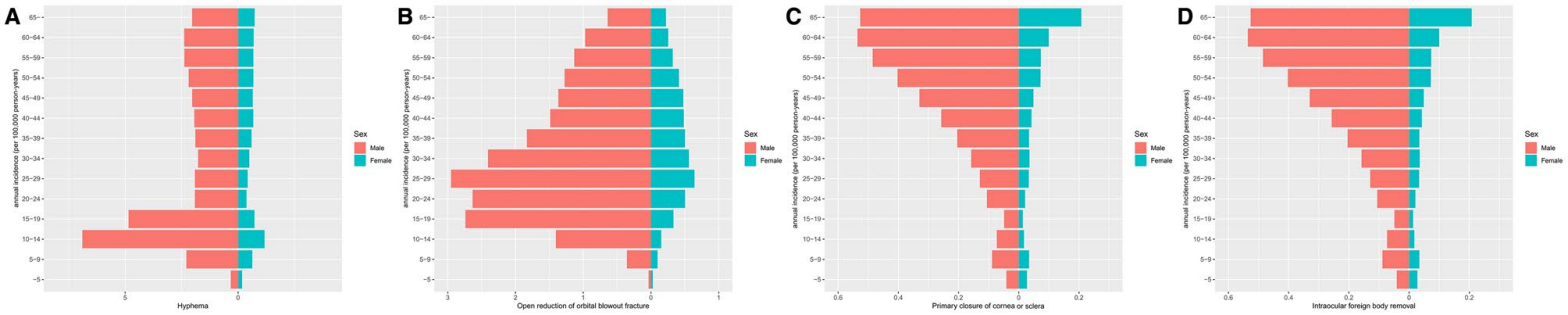
Comparisons of incidence rates by age distribution in males (red) and females (green). The highest incidence of hyphemas (**A**) was in patients aged 10–14 years, for both sexes, and the highest incidence of open reductions of orbital blowout fractures (**B**) was in patients aged 25–29 years, for both sexes. The incidence of open globe injuries (**C**, **D**) peaked in males at 60–64 years of age, whereas that of females increased with age.

From 2010 to 2018, the average annual incidence (per 100,000 person-years) of hyphemas in the Republic of Korea was 18.43 ± 5.17; the incidence gradually decreased from 2010 (28.60 per 100,000) to 2018 (12.82 per 100,000). The incidence was highest among those aged 10–14 years and was higher among males than among females (29.28 vs. 7.60 per 100,000 person-years; Table 1; Fig. 1A). The monthly numbers of hyphema patients showed significant seasonality, with peaks in June and September (amplitude: 174.81; p < 0.01; Fig. 2A). 

### Orbital blowout fracture

The average annual number of patients (per 100,000 person-years) who received BOF surgery between 2010 and 2018 was 11.58 ± 1.27. The incidence had a decreasing trend, from 13.64 per 100,000 in 2010 to 9.28 per 100,000 in 2018. The incidence was highest in patients aged 25–29 years, and was higher among males than among females (18.72 vs. 4.45 per 100,000 person-years; Table 1; Fig. 1B). The monthly numbers of BOF surgeries showed significant seasonality (amplitude: 23.17; p < 0.01), with peaks in May and October (Fig. 2B).

### Open globe injury

The average annual number of patients (per 100,000 person-years) treated with PCCS surgery between 2010 and 2018 was 1.99 ± 0.24, and that of IOFB removal was 0.39 ± 0.04. No distinct yearly trends were evident between 2010 and 2018. For PCCS, the annual incidence of surgery was 2.04 per 100,000 in 2010 and 1.99 per 100,000 in 2018. The average numbers of IOFB removals were 0.31 per 100,000 in 2010 and 0.38 per 100,000 in 2018. For both OGIs, the incidence rate of males was higher than that of females (PCCS: 3.15 per 100,000 person-years for males vs. 0.83 per 100,000 person-years for females; IOFB removal: 0.68 per 100,000 person-years for males vs. 0.09 per 100,000 person-years for females). Both OGIs also showed an increasing trend by age (Table 1; Fig. 1C,D). The monthly numbers of PCCS and of IOFB removals showed significant seasonality (PCCS: amplitude 11.96, p < 0.01; IOFB removal: amplitude: 6.72, p < 0.01); peaks appeared most frequently in September (Fig. 2C,D).

**Table 1:** Frequencies of major types of ocular trauma in Korea (2010–2018).

	Total number	Annual incidence (per 100,000 person-years) in average
**Hyphema**		
Total	84,133	18.43 ± 5.17
Male	66,757	29.28 ± 8.53
Female	17,376	7.60 ± 1.88
**Open reduction of BOF**		
Total	53,053	11.58 ± 1.27
Male	42,845	18.72 ± 2.32
Female	10,208	4.45 ± 0.30
**Primary closure of cornea or sclera**		
Total	9,140	1.99 ± 0.24
Male	7,252	3.15 ± 0.31
Female	1,888	0.83 ± 0.19
**IOFB removal**		
Total	1,779	0.39 ± 0.04
Male	1,561	0.68 ± 0.07
Female	215	0.09 ± 0.03

*BOF* blowout fracture, *IOFB* intraocular foreign body.

## Discussion

In this nationwide population-based study of ocular trauma, highest incidence rates were shown in hyphema, BOF, and OGI respectively in descending order. We confirmed the male predominance of all major types of ocular trauma^1, 13, 14^, and found clear seasonality in each type of trauma; however, the yearly trends, age distributions, and amplitudes of periodicities differed depending on the type of trauma.

To date, ocular trauma has been thought to occur most at relatively young ages^1, 11, 12, 15^, but we found different patterns of age distribution, depending on the type of ocular trauma: hyphema was most prevalent at 10–14 years of age, BOFs at 25–29 years of age, and OGIs at ≥ 60 years of age. Previous studies indicated that hyphema mainly occurred at the ages of 5–14 years during participation in sports, while BOFs occurred among those in their 20 s and 30 s as a result of violent assaults, traffic accidents, and sports^12, 16, 17^. We found a higher incidence of OGIs in relatively old patients, which was presumably due to deteriorated physical ability and delayed reaction to accidents.

Although all types of trauma showed significant seasonality, hyphemas (amplitude: 174.81) and BOFs (23.17) showed higher amplitudes compared to OGIs (PCCS: 11.96; IOFB removal: 6.72). Specifically, blunt trauma injuries including hyphema and BOF had peaks between May and October, a period associated with increased participation in outdoor activities. However, OGIs, including PCCS and IOFB removals, showed less seasonality, probably because of the high relevance of occupational accidents^18, 19^.

The incidence of hyphema and BOFs declined between 2010 and 2018. This may have been due to the development of safety measures in the Republic of Korea that included increased use of eye protection during outdoor activities. However, the incidence of OGIs did not decrease over the period. Furthermore, in contrast to closed globe injuries, OGIs can result in permanent visual impairment through endophthalmitis, traumatic cataracts, retinal detachment, and corneal opacity. Thus, increased efforts to prevent OGIs are needed.

We suggest that prevention strategies based on age or type of injuries to be considered. For pediatric ocular injuries, prevention system or education program to avoid accidents in potentially dangerous activities should be implemented^20^. Parents or teachers have to play an important role in supervision^6^. To reduce ocular injury by traffic accidents, enhancement of legal sanctions including enforcement of seatbelt laws or development of automotive technology for safety are required. Additionally, in workplace, efforts such as enforcing to wear preventive equipment or advancement of technology to reduce unintentional accidents should be made.

There were some limitations to this study. First, we could not identify the exact cause of each ocular trauma. Second, we inferred the incidence rates of BOFs and OGIs, based on the numbers of surgeries. Thus, the incidence rates of BOFs and OGIs may have been underestimated. However, our data for 50 million Korean residents with a single NHI system with affordable medical costs likely reflected actual incidence rates, with minimal chance of selection bias^21^.

In conclusion, in this nationwide population-based study, we investigated the incidence rates and seasonality of four major types of ocular trauma in the Republic of Korea from 2010 to 2018. All of them showed male predominance, but each showed different age distributions. While the incidence of blunt trauma injuries such as hyphemas and BOFs decreased, that of OGIs did not decrease. Based on these results, more research is needed to determine the causes of ocular trauma and to establish appropriate prevention policies.

## Methods

### Study design and database

We conducted a nationwide, population-based, cross-sectional study using the Korean NHI claims database for January 2010 to December 2018. This study was approved by the Institutional Review Board of St. Vincent’s Hospital (VC19ZESI0179). All Korean residents must enroll in the NHI system, so this database encompasses all medical claims in the Republic of Korea. We identified all patients who received a principal diagnosis or corresponding surgery in one of the following: hyphema (International Classification of Diseases, 10th revision code H210), reduction of orbital BOF (S5211), PCCS (S5380), and IOFB removal (S4891 or S4892). In cases of hyphema, only newly diagnosed cases were included in the study. The numbers of patients diagnosed or treated with each disorder each month and year were collected.

### Statistical analysis

We calculated incidence rates depending on year, sex, and age group, and determined the differences among them. We acquired incidence rate based on census population data of each group obtained from Statistics Korea. Mann–Whitney U-test was employed to compare average annual incidence rates between male and female. In addition, the cosinor test was used to examine seasonal variation based on monthly incidence rates^22^. All analyses were conducted using R software, version 3.6.1 (R: A Language and Environment for Statistical Computing, R Core Team, R Foundation for Statistical Computing, Vienna, Austria (2019) https://www.R-project.org).

## Data Availability

The datasets generated during and/or analysed during the current study are available from the corresponding author on reasonable request.
